# Age-related ultrastructural changes in spheroids of the adipose-derived multipotent mesenchymal stromal cells from ovariectomized mice

**DOI:** 10.3389/fncel.2023.1072750

**Published:** 2023-02-17

**Authors:** Vitalii Kyryk, Oleg Tsupykov, Alina Ustymenko, Ekaterina Smozhanik, Iryna Govbakh, Gennadii Butenko, Galyna Skibo

**Affiliations:** ^1^Cell and Tissue Technologies Department, Institute of Regenerative Medicine, National Scientific Center M.D. Strazhesko Institute of Cardiology, Clinical and Regenerative Medicine of the National Academy of Medical Sciences of Ukraine, Kyiv, Ukraine; ^2^Laboratory of Pathophysiology and Immunology, D. F. Chebotarev State Institute of Gerontology of the National Academy of Medical Sciences of Ukraine, Kyiv, Ukraine; ^3^Department of Cytology, Bogomoletz Institute of Physiology of the National Academy of Sciences of Ukraine, Kyiv, Ukraine; ^4^Department of General Practice-Family Medicine, Kharkiv Medical Academy of Postgraduate Education, Kharkiv, Ukraine

**Keywords:** ultrastructure, spheroids, adipose-derived multipotent mesenchymal stromal cells, ovariectomy, aging

## Abstract

**Introduction:** Adipose-derived multipotent mesenchymal stromal cells (ADSCs) are widely used for cell therapy, in particular for the treatment of diseases of the nervous system. An important issue is to predict the effectiveness and safety of such cell transplants, considering disorders of adipose tissue under age-related dysfunction of sex hormones production. The study aimed to investigate the ultrastructural characteristics of 3D spheroids formed by ADSCs of ovariectomized mice of different ages compared to age-matched controls.

**Methods:** ADSCs were obtained from female CBA/Ca mice randomly divided into four groups: CtrlY—control young (2 months) mice, CtrlO—control old (14 months) mice, OVxY—ovariectomized young mice, and OVxO—ovariectomized old mice of the same age. 3D spheroids were formed by micromass technique for 12–14 days and their ultrastructural characteristics were estimated by transmission electron microscopy.

**Results and Discussion:** The electron microscopy analysis of spheroids from CtrlY animals revealed that ADSCs formed a culture of more or less homogeneous in size multicellular structures. The cytoplasm of these ADSCs had a granular appearance due to being rich in free ribosomes and polysomes, indicating active protein synthesis. Extended electron-dense mitochondria with a regular cristae structure and a predominant condensed matrix were observed in ADSCs from CtrlY group, which could indicate high respiratory activity. At the same time, ADSCs from CtrlO group formed a culture of heterogeneous in size spheroids. In ADSCs from CtrlO group, the mitochondrial population was heterogeneous, a significant part was represented by more round structures. This may indicate an increase in mitochondrial fission and/or an impairment of the fusion. Significantly fewer polysomes were observed in the cytoplasm of ADSCs from CtrlO group, indicating low protein synthetic activity. The cytoplasm of ADSCs in spheroids from old mice had significantly increased amounts of lipid droplets compared to cells obtained from young animals. Also, an increase in the number of lipid droplets in the cytoplasm of ADSCs was observed in both the group of young and old ovariectomized mice compared with control animals of the same age. Together, our data indicate the negative impact of aging on the ultrastructural characteristics of 3D spheroids formed by ADSCs. Our findings are particularly promising in the context of potential therapeutic applications of ADSCs for the treatment of diseases of the nervous system.

## Introduction

Adipose tissue is a rich source of multipotent mesenchymal stromal cells (ADSCs). The use of ADSCs in modern medicine makes it possible to realize their multilinear potential in various pathological conditions: in the treatment of diseases of the nervous system (Ma et al., 2020; Zhou et al., 2020), endothelial dysfunction in critical limb ischemia and diabetes mellitus (Magenta et al., 2021), endocrine dysfunction (Amer et al., 2018), in coronary heart disease (Murohara et al., 2009), in plastic and reconstructive surgery of soft tissues, and the musculoskeletal system, etc.

However, it is important to note that the therapeutic success of cell therapy may depend on many factors, including the age of the donor. In a study by Liu et al. (2017), a negative effect of the age of the donor’s adipose tissue on the quantity and quality of human mesenchymal adipose-derived cells was shown: both the number of colony-forming units of fibroblasts and the number of cells obtained from the stromal-vascular fraction *in vitro*, as well as the rate of their proliferation, are reduced. In addition, a violation of the migration ability of cells obtained from old donors was observed, which is explained by the reduced expression of chemokine receptors such as CXCR4 and CXCR7 (Liu et al., 2017).

Most of the pathological conditions that require cell therapy using autologous stem cells occur mainly in the elderly, so it is relevant to establish criteria for the biological safety of stem cells of adipose origin obtained in the menopausal and postmenopausal periods, which are accompanied by estrogen deficiency (Eastell et al., 2016).

The features of ADSCs are affected by both culture conditions and intercellular signals. Unlike 2D cultivation, three-dimensional (3D) culture of ADSCs in the form of spheroids, which partially mimics the conditions of the microenvironment (niche) of stem cells, can significantly improve their survival in the recipient tissue and increase the overall regenerative potential (Egger et al., 2018).

The aim of our study was to investigate the ultrastructural characteristics of multicellular three-dimensional spheroids formed by ADSCs obtained under conditions of estrogen deficiency in a model of ovariectomy in mice of different ages compared to age-matched controls. Our study aimed at establishing the mechanisms of cellular self-organization, contact intercellular signaling, extracellular matrix production, and resistance to hypoxia depending on the size of the spheroid.

## Materials and methods

All animal procedures were performed in accordance with “European Convention for the protection of Vertebrate Animals Use for Experimental and Other Scientific Purposes” (Strasbourg, 1986), “European Directive 2010/63/EU on the protection of animals used for scientific purposes” and the Law of Ukraine “On protection of animals from cruelty” as well as in abidance to all principles of bioethics and biosafety regulations. The study was approved by Ethics Committee of the Institute of Genetic and Regenerative Medicine (protocol no. 9-2021 dated December 15, 2021).

### Animals

Adipose tissue was obtained from young (2 months) and old (14 months) female CBA/Ca mice, which were kept under standard conditions in a vivarium of the D. F. Chebotarev State Institute of Gerontology NAMS of Ukraine under a 12:12 h light/dark cycle with access to water and food *ad libitum* (Table 1).

**Table 1:** The distribution of animals in the experimental groups.

Experimental group	Young	Old
	The age of animals at the time of surgery (months); number (n)	
Control, sham-operated (Ctrl)	2 months (*n* = 8)	14 months (*n* = 11)
Ovariectomy (OVx)	2 months (*n* = 7)	14 months (*n* = 8)

### Ovariectomy modeling

Animals were anesthetized by intraperitoneal administration of 2.5% solution of 2,2,2-tribromethanol (Sigma-Aldrich, St. Louis, MO, USA) at a dose of 400 mg/kg and bilateral ovariectomy was performed under aseptic conditions using microsurgery technique. The animals of the same age which had only incisions of the abdominal cavity and isolation of the ovaries without resection (sham-operated) were used as a control group. Wounds were sutured in layers; the animals were kept under a heat lamp until the recovery from anesthesia.

### Adipose-derived mesenchymal stromal cells isolation and culture

Murine ADSCs cultures were obtained and characterized according to standard methods (Yu et al., 2011). Two months after ovariectomy the CBA/Ca mice were euthanized by cervical dislocation under the anesthesia with 2.5% solution of 2,2,2-tribromethanol at a dose 400 mg/kg. Under sterile conditions, subcutaneous adipose tissue was isolated, minced with scissors into 1 mm^3^ pieces in DMEM/F12 medium (Sigma-Aldrich, St. Louis, MO, USA) and incubated in 0.1% solution of collagenase type IA (Sigma-Aldrich, St. Louis, MO, USA) for 60 min at 37°C with constant stirring on a shaker at 100 rpm. The resulting cell suspension was washed in 10 ml DMEM medium (Sigma-Aldrich, St. Louis, MO, USA) by centrifugation at 300× *g* for 5 min. The supernatant with mature adipocytes and debris was discarded and pellet passed through a sterile cell strainer with a pore diameter of 100 μm (Greiner bio-one, Kremsmünster, Austria). Cells of the stromal-vascular fraction were cultured in a CO_2_ incubator in humidified atmosphere with 5% CO_2_ at a temperature of +37°C in complete nutrient medium DMEM-LG (Sigma-Aldrich, St. Louis, MO, USA) supplemented with 15% fetal bovine serum (FBS) (HyClone Laboratories Inc., South Logan, UT, USA), penicillin 100 U/ml, streptomycin 100 μg/ml (Sigma-Aldrich, St. Louis, MO, USA), 1:100 nonessential amino acids (Sigma-Aldrich, St. Louis, MO, USA). The nutrient medium was replaced in 3 days. Cells were sub-cultured to achieve 80% monolayer confluency (for 4–5 days) using 0.25% trypsin (Sigma-Aldrich, St. Louis, MO, USA) and 0.02% Versene solution (Bio-Test Laboratory, Kyiv, Ukraine).

### Immunophenotyping of ADSCs

On the 2nd passage, cells were analyzed by flow cytometry with BD FACSAria cell sorter (Becton Dickinson, Franklin Lakes, NJ, USA) using anti-mouse monoclonal antibodies: CD90 APC-Cy7 (BD Biosciences, cat. no. 561401, Franklin Lakes, NJ, USA), CD105 APC (Invitrogen, cat. no. 17-1051-82, Carlsbad, CA, USA), CD73 PE (BD Biosciences, cat. no. 550741, Franklin Lakes, NJ, USA), CD44 PE (BD Biosciences, cat. no. 553134, Franklin Lakes, NJ, USA), CD45 PE (Thermo Fisher Scientific, cat. no. MA1-10233, Waltham, MA, USA), CD34 Alexa Fluor^®^ 647 (BD Biosciences, cat. no. 560230, Franklin Lakes, NJ, USA).

### Three-dimensional spheroids formed by ADSCs

After the second passage, the monolayer culture of ADSCs was transferred to a suspension state, washed from the enzyme, and 1.5 × 10^6^ cells were transferred to a 5 ml PS tube with a non-adhesive surface. After the formation of the spheroids (after 24–48 h of culturing in the tube), the cells were cultured for 12–14 days in a CO_2_ incubator under conditions of humidified atmosphere with 5% CO_2_ at a temperature of +37°C. The nutrient medium was completely replaced every 2–3 days. On the 5–7th day, the standard medium for cultivation was replaced with medium for adipogenic induction to confirm that the obtained cultures met the minimal criteria to define ADSCs in terms of potential for directed differentiation. Adipogenic differentiation was performed using DMEM High Glucose medium (Gibco, USA) supplemented with 10% FBS, dexamethasone (1 μM), indomethacin (200 μM), 3-Isobutyl-1-methylxanthine (500 μM), and insulin (5 μg/ml), all reagents—Sigma-Aldrich, St. Louis, MO, USA. The medium was changed every 3 days and the cells were cultured for 14 days. The area of spheroids was calculated according to the formula: *S* = 4▪πR^2^, where π is a constant of 3.14; R is the radius of the sphere. To estimate the area of spheroids, we used images obtained under inverted microscope IX-71 (Olympus, Japan) using a DP20 (Olympus, Japan) camera and performed measurements using QuicjPHOTO MICRO 2.3 software (Olympus, Japan). On day 14 *in vitro* grown cultures (DIV14) ADSCs spheroids were rinsed with PBS and fixed with 4% formaldehyde. After staining with Oil Red O (Sigma-Aldrich, St. Louis, MO, USA), the cells were visualized with light microscopy IX-71 (Olympus, Japan).

### Transmission electron microscopy (TEM)

The ADSCs spheroids from different experimental groups were centrifuged at 500× *g* and pellets were processed for TEM according to usual protocols (Tsupykov et al., 2016). Briefly, the culture medium was replaced with 4% formaldehyde and 2.5% glutaraldehyde (Fluka, Buchs, Switzerland) in 0.1 M phosphate buffer (PB). ADSCs spheroids were then rinsed, postfixed in 1% osmium tetroxide (Sigma-Aldrich, St. Louis, MO, USA) in 0.1 M PB, dehydrated in ascending concentrations (50%–100%) of ethanol, and then embedded in Epon 812 (Fluka, Buchs, Switzerland). Ultrathin sections (50–70 nm) were cut with a diamond knife, collected on single slot grids and then counterstained with lead citrate (Fluka, Buchs, Switzerland) and alcoholic uranyl acetate (Merck, Darmstadt, Germany). Grids were examined in JEOL 100-CX (JEOL, Japan) electron microscope operating at 80 kV.

## Results

### Immunophenotyping of adipose-derived multipotent mesenchymal stromal cells cultures

**Figure 1:**
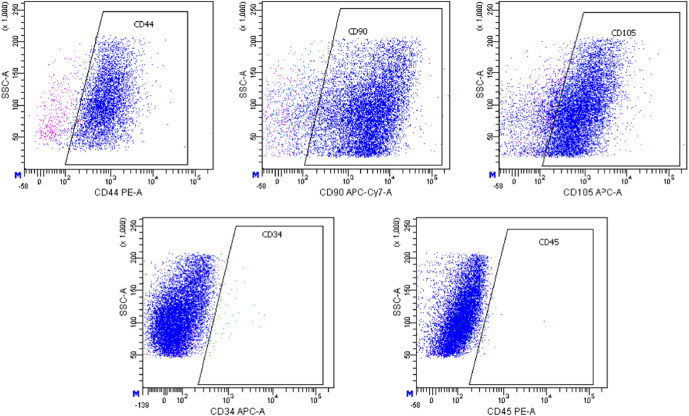
Histograms of expression of CD44, CD90, CD105, CD34, and CD45 markers in the culture of murine ADSCs from young animals according to flow cytometry, the 2nd passage.

According to flow cytometry data, the ADSCs cultures obtained from both young and old mice expressed typical stromal markers CD44, CD90, and CD105 at a high level (≥95%). At the same time, low expression of hematopoietic markers CD34 and CD45 (<3%) was noted (Figure 1). In our previous study, we confirmed the potential of ADSCs from ovariectomized mice to differentiate into osteogenic and adipogenic directions (Ustymenko et al., 2019). Taking into account significant changes in adipose and bone tissue associated with hormonal changes in old age, we focused our research efforts specifically on assessing the osteogenic and adipogenic differentiation potential of adipose-derived cells. As a result, the morphology, phenotype and differentiation potential of obtained ADSCs cultures meet the minimal criteria for defining multipotent mesenchymal stromal cells according to the International Society for Cellular Therapy (ISCT) position statement (Dominici et al., 2006).

### Three-dimensional spheroids formed by ADSCs

**Figure 2:**
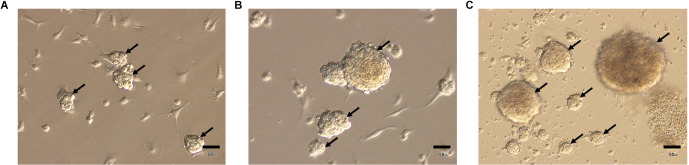
Three-dimensional spheroids (arrows) formed by ADSCs, obtained from young **(A)**, old **(B)**, and ovariectomized old **(C)** animals. Light microscopy. Scale bar—50 μm.

Our observations showed that ADSCs obtained from control young animals form a culture of more or less homogeneous spheres in size, the surface area of which ranges approximately from 5,000 μm^2^ to 7,000 μm^2^ (Figure 2A).

At the same time, ADSCs obtained from control and ovariectomized old animals are able to form a 3D-culture heterogeneous in size, the surface area of which approximately ranges from 3,000 μm^2^ to 160,000 μm^2^ (Figures 2B,C).

In addition, spheroids formed by ADSCs obtained from control and ovariectomized old animals exhibit enhanced adipogenic potential compared to ADSCs obtained from young mice (data not shown).

### Ultrastructural analysis of spheroids from ADSCs

**Figure 3:**
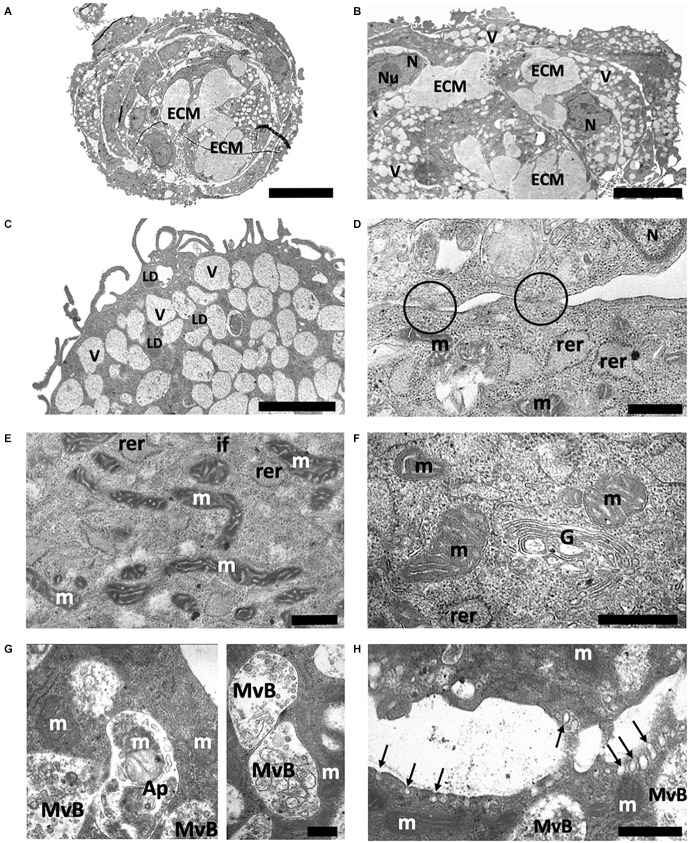
Structure of spheroids from the adipose-derived multipotent mesenchymal stromal cells from young control CBA/Ca mice. **(A)** Central section giving an overview of representative ADSCs spheroid after 14 days of culture. Spheroid consists of the dense outer zone and the inner area, where round or polygonal cells are embedded in an extracellular matrix (ECM). **(B)** Elongated cells of the outer area and a part of the inner zone of ADSCs spheroids with an extracellular matrix. The ADSCs cytoplasm is extremely rich in vacuoles (V). N—nuclei; Nu—nucleolus. **(C)** In the outer area ADSCs has many thin pseudopodia extended from the cell surface. The ADSCs cytoplasm contains a large number of vacuoles and a moderate amount of lipid droplets (LD). **(D)** Plasma membranes of adjacent adipose-derived multipotent mesenchymal stromal cells are attached by numerous junctions, which appeared as tight junctions (circles). **(E)** Detailed view of cytoplasm containing a relative high amount of elongated electron-dense mitochondria (m), rough endoplasmic reticulum (rer), and intermediate filament (if) bundles. **(F)** Well-developed Golgi apparatus (G) producing large secreting granules. **(G)** ADSC cytoplasm containing a number of late endosomal multivesicular bodies (MvB). A fragment of mitochondria is observed inside the autophagosome (Ap). **(H)** The sites of active exocytosis (arrows) on the plasma membrane. Scale bars: **(A)**—25 μm, **(B)**—15 μm, **(C)**—2 μm, **(D,E,H)**—1 μm, **(F,G)**—0.5 μm.

Results from electron microscopy showed that adipose-derived multipotent mesenchymal stromal cells (ADSCs) from young control CBA/Ca mice under non-adhesive conditions formed a culture of more or less homogeneous in size multicellular structures (from 75 μm to 95 μm in diameter)—spheroids consisted of several surface layers and an inner zone (Figures 3A,B).

The inner zone consisted of polygonal or round cells, embedded in an extracellular matrix (Figure 3B). These cells were tightly packed, with narrow extracellular spaces. In the inner area, ADSCs were attached to each other by numerous junctions, which, at high magnification, appeared as tight junctions (Figure 3D). ADSCs had a single, irregularly shaped and large euchromatic nuclei with one or more nucleoli (Figures 3B,C). Elongated electron-dense mitochondria with a regular cristae structure and a predominant condensed matrix were observed in ADSCs from CtrlY group, which could indicate high respiratory activity (Figure 3E). The cytoplasm had a granular appearance due to being rich in free ribosomes and polyribosomes, indicating active protein synthesis (Figures 3E,F). Interestingly, the rough endoplasmic reticulum cisternae were often dilatated and contained moderately electron-dense material (Figures 3D–F). Furthermore, transmission electron microscopy showed well-developed Golgi apparatus in the juxta-nuclear area producing large secreting granules (Figure 3F). A number of multivesicular bodies, a special kind of late endosomes, were also observed in ADSC cytoplasm (Figure 3G). Multivesicular bodies were very heterogeneous in size and morphology. Whole intracellular elements such as mitochondria were sequestered inside an autophagosome that then fused with multivesicular bodies to form amphisome (Figure 3G). Electron microscopy analysis revealed the process of active exocytosis on the plasma membrane of ADSCs (Figure 3H).

**Figure 4:**
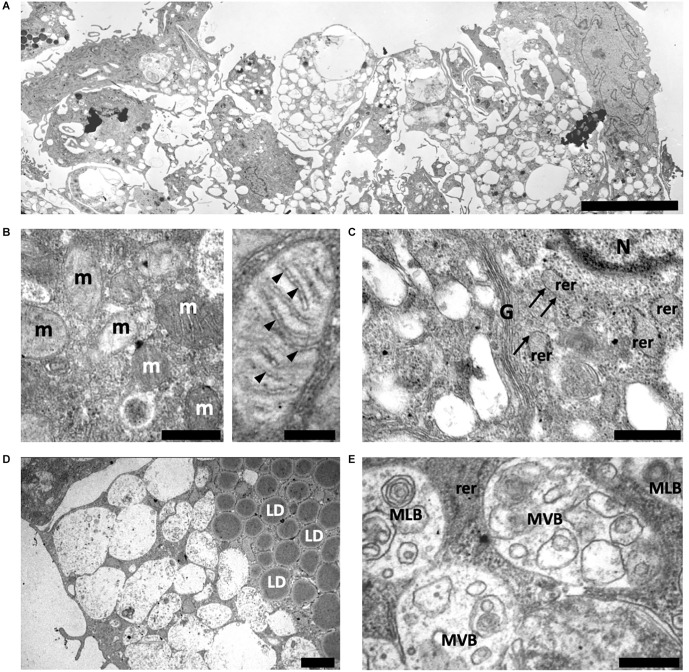
Structure of spheroids of the adipose-derived multipotent mesenchymal stromal cells from old control CBA/Ca mice. **(A)** An overview of representative ADSCs spheroid after 14 days of culture. ADSCs are not tightly packed in spheroid and had a wide intercellular space. **(B)** Left electronogram—The mitochondrial population is heterogeneous and is represented by mitochondria with both electron-dense condensed and enlightened matrix. Right electronogram—A detailed view of round mitochondrion with enlightened matrix and cristae (arrowheads) arranged perpendicular to the mitochondrial tubular axis. **(C)** The ADSCs cytoplasm contains rough endoplasmic reticulum (rer) and well-developed Golgi apparatus (G). Areas of rough endoplasmic reticulum that do not contain ribosomes are marked with arrows. N—nuclei. **(D)** A large number of lipid droplets (LD) is observed in the ADSCs cytoplasm. **(E)** Typical multivesicular (MVB) and multilamellar bodies (MLB) are observed in the ADSCs cytoplasm. Scale bars: **(A)**—15 μm, **(B)** left—0.5 μm, right—0.2 μm, **(C,D)**—1 μm, **(E)**—0.5 μm.

At the same time, ADSCs from old control mice (CtrlO group) formed a culture of heterogeneous in size spheroids (from 60 μm to 370 μm in diameter). Unlike spheroids formed by ADSCs from young animals, ADSCs from old mice were not tightly packed and had a wide intercellular space (Figure 4A). It was also difficult to clearly separate the outer and inner layers, as was observed in spheroids obtained from young mice.

Transmission electron microscopy of ADSCs from CtrlO group showed a similar ultrastructural architecture to young control, but there were some noticeable differences. In these ADSCs the mitochondrial population was heterogeneous, a significant part was represented by more round structures (Figure 4B). This may indicate an increase in mitochondrial fission and/or an impairment of the fusion. Mitochondria with both electron-dense condensed and enlightened matrix were presented, denoting their different respiratory activity (Figure 4B). Significantly fewer polysomes and ribosomes attached to the surface of rough endoplasmic reticulum were also observed in the cytoplasm of ADSCs from CtrlO group, indicating low protein synthetic activity (Figure 4C). In addition, the cytoplasm of ADSCs in spheroids from old mice had notably increased amounts of lipid droplets compared to cells obtained from young animals (Figure 4D). The cytoplasm was extremely rich in endosomal elements showing typical multilamellar and multivesicular structures (Figure 4E).

**Figure 5:**
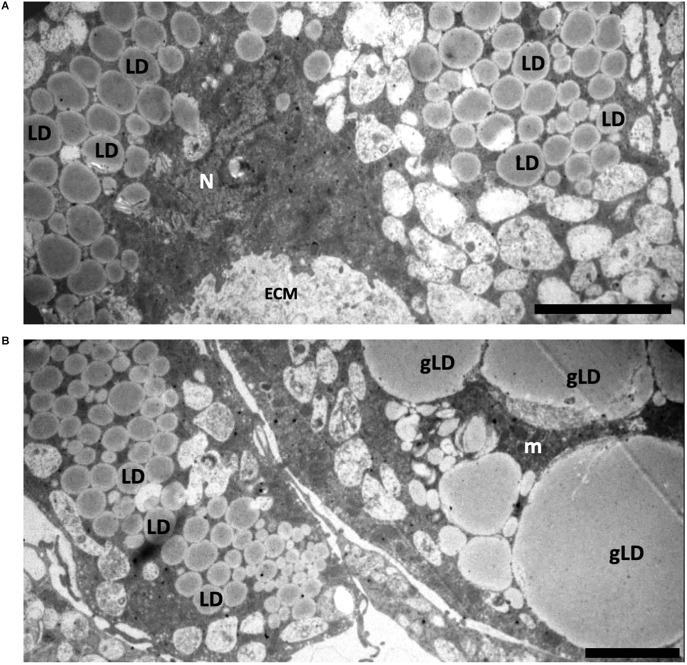
Structure of spheroids of the adipose-derived multipotent mesenchymal stromal cells from young **(A)** and old **(B)** ovariectomized CBA/Ca mice. **(A)** The cytoplasm of ADSCs from young ovariectomized mice have a large number of lipid droplets (LD) and endosomal elements. ECM—extracellular matrix. N—nuclei. **(B)** The cell cytoplasm of ADSCs from old ovariectomized mice contains giant lipid droplets (gLD). m—mitochondria. Scale bars: **(A,B)**—2 μm.

Electron microscopic analysis showed that ovariectomy led to changes in the ultrastructure of ADSCs in spheroids. The cytoplasm of ADSCs from young ovariectomized mice had significantly increased amounts of lipid droplets and endosomal elements compared to cells obtained from young control animals (Figure 5A).

Interestingly, the most prominent ultrastructural feature of the ADSCs from old ovariectomized mice was giant lipid droplets (3–4.5 μm in diameter) that probably formed by fusion or coalescence of smaller adjacent lipid droplets (Figure 5B). The cytoplasm was also characterized by numerous vacuolar elements.

## Discussion

The issue of the impact of aging on the phenotypic and functional characteristics of stem cells is quite relevant for modern regenerative medicine. Choosing the optimal source of cells, assessing their quality, taking into account the age and health status of the donor, determine the overall effectiveness and safety of cell therapy.

The aging is associated with altered immune and metabolism dysfunctions, increased inflammation and significant changes in the physiological levels of sex hormones. Multiple pathogenic pathways induce defective adipogenesis, inflammation, aberrant adipocytokine production, and insulin resistance, leading to age-related adipose tissue dysfunction with the functional decline of adipocyte progenitors and accumulation of senescent cells (Ou et al., 2022). Under ovariectomy conditions in mice, the proliferative capacity and osteogenic potential of ADSCs are significantly impaired compared to normal animals (Wang et al., 2017). In mice after ovariectomy, enhanced adipogenic differentiation of ADSCs is likely to be the important cause for increased adipogenesis *in vivo* and subsequent obesity-like changes in body mass (Fu et al., 2014). The regenerative potential of ADSCs in conditions of age-related estrogen deficiency is also impaired. In particular, ADSCs from aged estrogen-deficient ovariectomized rats have less capacity to increase tenocyte proliferation and healing in indirect co-culture system compared with normal ADSCs (Veronesi et al., 2015).

In our previous study, it was demonstrated that there were no statistically significant differences in the expression of all typical surface markers of ADSCs in young and old mice (Ustymenko et al., 2019). This fact indicates that the immunophenotype of ADSCs and, probably, their quantity in adipose tissue do not change with age, that confirm in studies of both human and animal samples published by other researchers.

In the study of Li et al. (2021) there were no significant differences in most of the surface markers between ADSCs from 1-month-old or 20-month-old mice, implying that the adipose tissue obtained from young animals had a comparative yield of ADSCs to that of old mice under the same conditions. No significant differences were found in the expression of surface markers on ADSCs from rats aged 2-, 9- and 24 months in the study of Muñoz et al. (2020). At the same time, ADSCs derived from horses older than 5 years old exhibited several molecular alternations which markedly limit their regenerative capacity. Aged ADSCs were characterized by increased gene expression of pro-inflammatory cytokines and miRNAs (IL-8, IL-1β, TNF-α, miR-203b-5p, and miR-16-5p), as well as apoptosis markers (p21, p53, caspase-3, caspase-9; Alicka et al., 2020).

Liu et al. (2017) showed while human ADSCs from different age populations are phenotypically similar, they present major differences at the functional level. Advancing age was found to have a significant negative effect on the adipogenic and osteogenic differentiation potentials of human ADSCs (Liu et al., 2017). Zhang et al. (2011) suggested that advanced age and comorbidity do not negatively impact isolation of ADSCs, and these stem cells retain significant capacity to acquire key endothelial cell phenotype throughout life. Chen et al. (2012) found that the doubling time of ADSCs from both age groups was maintained below 70 h and authors concluded that the proliferation and osteogenic differentiation of ADSCs were less affected by age and multiple passages than in bone marrow-derived MSCs cultures.

The authors explain impairment of proliferative and differentiation potential by the existence of internal changes in ADSCs during aging, which is associated with senescence associated secretory phenotype (SASP) during chronic inflammation and metabolic disorders. The components of the pathologic secretory phenotype are quite heterogeneous and may depend on the cell type. In particular, it has been shown that increased levels of certain cytokines, chemokines and growth factors (IL-4, IL-13, IL-17, CCL3, CCL25 and GM-CSF) can characterize the SASP profile for ADSCs from elderly donors (Li et al., 2021).

The 3D cultivation of ADSCs in the form of spheroids, which partially simulates the conditions of the stem cells’ micro-environment (niche), can significantly improve their survival in the recipient’s tissue and increase the overall regenerative potential. The study of the ultrastructural characteristics of cells in the 3D spheroids is aimed at establishing the mechanisms of cell self-organization, contact intercellular signaling, production of the extracellular matrix, resistance to hypoxia, depending on the size of the spheroid.

It has been shown that 3D spheroid culture reduces size by increasing the secretion of extracellular vesicles. This event is mediated by a decrease in actin polymerization (F-actin). Probably, the large size of ADSCs spheroid cultures from old animals in our study indicates a violation of the ability to release microvesicles into the extracellular space (Mo et al., 2018).

In the study of Li et al. (2022) scanning electron microscopy showed that surfaces of spheroids formed in simulated microgravity culture system were relatively smooth and organized in a regular, granular shape, which may be beneficial for ever exchange of nutrients and gases evenly. Lee et al. (2021) proposed method of hybridization of ADSCs spheroids with polydopamine coated single-segmented fibers to enhance viability regardless of sizes and increase their functionality by regulating the size of spheroids. Transmission electron microscopy images showed that cell-only spheroids exhibited disintegrated membranes and empty spaces. In contrast, the cell membranes in fiber incorporated spheroids were tightly bound with each other, and disconnected or empty regions were minimal (Lee et al., 2021).

Step-by-step directed differentiation of multipotent cells in 3D spheroids *in vitro* will allow to partially reproduce the physiological mechanisms of tissue formation *in vivo* and to obtain a spatially organized culture of cells capable of survival, proliferation, and differentiation into certain direction.

Baraniak and McDevitt (2012) showed that cell proliferation and differentiation potential of dissociated cells retrieved from spheroids of mesenchymal stem cells were compared to conventional adherent monolayer cultures. Cells that had been cultured within spheroids recovered morphology typical of cells cultured continuously in adherent monolayers and retained their capacity for multi-lineage differentiation potential. In fact, more robust matrix mineralization and lipid vacuole content were evident in recovered MSCs when compared to monolayers, suggesting enhanced differentiation by cells cultured as 3D spheroids (Baraniak and McDevitt, 2012).

Laschke et al. (2014) discovered that scaffolds seeded with osteogenic differentiated spheroids exhibited a markedly impaired vascularization caused by the lost ability of differentiated spheroids to form microvascular networks. This was associated with a reduced tissue incorporation of the implants and indicating the dedifferentiation of the spheroids under the given *in vivo* conditions. These findings indicate that osteogenic differentiation of ADSCs spheroids markedly impairs their vascularization capacity (Laschke et al., 2014).

In our previous study, it was shown that 3D grafts of ADSCs cultured in spheroids are able to improve bone tissue regeneration in a model of bone injury in mice. The grafts previously differentiated into osteogenic direction provide better morphological indicators of bone recovery, compared with the spheroids without prior differentiation. Intensive migration of cells from spheroids to an adhesive surface *in vitro* proves the ability of cells to survive in 3D culture. At the same time, the morphology of cells on the surface of spheroids under the influence of osteoinductive differentiation factors changes and proliferative activity decreases (Kyryk et al., 2022).

## Conclusion

Thus, we can suggest that ADSCs throughout life retain a significant amount in adipose tissue and a high functional potential *in vitro*, which can be effectively used in cell therapy strategies especially in elderly patients. At the same time, our data indicate the negative impact of ovariectomy on the ultrastructural characteristics of 3D spheroids formed by ADSCs. Our findings are particularly promising in the context of vigilance for potential therapeutic applications of ADSCs from old donors.

## Data availability statement

The raw data supporting the conclusions of this article will be made available by the authors, without undue reservation.

## Ethics statement

The animal study was reviewed and approved by the Ethics Committee of the Institute of Genetic and Regenerative Medicine (protocol no. 9-2021 dated December 15, 2021) and performed in accordance with the European Union Directive of 22 September 2010 (2010/63/EU) for the protection of animals used for scientific purposes.

## Author contributions

VK, GB, and GS: conceptualization. VK, AU, ES, IG, and OT: data collection and analysis. VK, AU, OT, and IG: writing—original draft preparation. VK, GB, and GS: writing—review and editing. ES: visualization. All authors contributed to the article and approved the submitted version.
